# Fundamental of swapping phenomena in naturally occurring gas hydrates

**DOI:** 10.1038/s41598-018-34926-2

**Published:** 2018-11-08

**Authors:** Avinash V. Palodkar, Amiya K. Jana

**Affiliations:** Energy and Process Engineering Laboratory, Department of Chemical Engineering, Indian Institute of Technology, Kharagpur, 721302 India

## Abstract

Amount of natural gas contained in the gas hydrate accumulations is twice that of all fossil fuel reserves currently available worldwide. The conventional oil and gas recovery technologies are not really suitable to gas hydrates because of their serious repercussions on geo-mechanical stability and seabed ecosystem. To address this challenge, the concept of methane-carbon dioxide (CH_4_-CO_2_) swapping has appeared. It has the potential in achieving safe and efficient recovery of natural gas, and sequestration of CO_2_. By this way, the energy generation from gas hydrates can become carbon neutral. This swapping phenomenon has not yet been elucidated at fundamental level. This work proposes a theoretical formulation to understand the physical insight into the transient swapping between natural gas and CO_2_ occurred under deep seabed and in permafrost. Addressing several practical concerns makes the model formulation novel and generalized enough in explaining the swapping phenomena at diverse geological conditions.

## Introduction

Naturally occurring gas hydrates are ice-like non-stoichiometric solid compounds, which are composed of rigid cages of water molecules that enclose natural gas molecules^1^. These gas hydrates are typically a global phenomenon in that unlike the conventional petroleum reserves, they are distributed worldwide in permafrost regions (130–2000 m below the ground) and along the most continental margins under deep seabed (800–3000 m below the seabed)^2,3^. It is estimated that the global volume of technically recoverable natural gas from hydrate deposits is in the order of 3 × 10^13^ cubic meter^4^, which could meet mankind energy needs for a couple of centuries.

Gas hydrates are relevant as the potential (i) energy resource for several 100 years, (ii) factor in global warming since methane escaping seems to occur from dissociated hydrates, and (iii) submarine geohazard, leading to possible catastrophic slope failure and marine ecosystem damage^5^. Importantly, the immense energy locked in hydrate deposits is mostly in the form of natural gas. This gas is relatively clean-burning premium fuel, and its market is growing exponentially because of the change of the world’s energy portfolio and infrastructure into a gas-based economy^6^.

In this light, the concept of CH_4_-CO_2_ swapping has appeared^7^. This technique is first subjected to techno-economic feasibility^4^ mainly for the enhanced recovery of next generation energy and carbon dioxide sequestration, indicating a way of catching two birds with one stone. Indeed, this replacement process has a promising role in dealing with (i) the effects of climate change expected to be caused by anthropogenic CO_2_ emission, (ii) geologic hazard and (iii) natural gas seepage through the crust. At this point one should note the recent experimental observation that the inclusion of N_2_ with CO_2_ leads to a reasonable improvement in replacement efficiency^4^.

Although there is some progress made on thermodynamic modeling of gas hydrates, a limited advancement has been noticed in kinetic formulation. In this regard, a couple of models are there for hydrate formation with pure water (e.g.^8–10^) having porous media (e.g.^11,12^). The effect of salt ions on hydrate kinetics is also taken into account (e.g.^13–15^). Recently, the gas swapping in clathrate hydrate is formulated^16^. They consider the isolation of the kinetic guest molecule exchange process from additional hydrate formation and mechanical changes to the hydrate bearing sand.

This swapping process simultaneously involves natural gas hydrate (NGH) decomposition and reformation followed by growth of a mixed hydrate. For this, the initial hydrate-gas equilibrium gets transformed to a new state of equilibrium. Understanding the physical insight into the process of simultaneous hydrate decay and formation involving the exchange of multiple guest gases that are basically methane and CO_2_ (pure or mixed) requires a generalized theory. Importantly, it should be versatile enough in predicting the transient state of the CH_4_-CO_2_ and –mixed CO_2_ exchange occurred within a porous cavity formed by pure/saline water molecules in the permeable sediments. There is no generalized model available to provide in-depth analysis of this multicomponent swapping process. With this research gap, this work proposes a theoretical formulation addressing various issues of practical importance for fundamental understanding of the naturally occurring gas hydrate phenomena in permafrost zones and under sea floor. Experimental data are used at diverse geological conditions to validate the formulation.

## Results

### Theory of gas swapping in hydrate lattice

A rigorous model is formulated for fundamental understanding of the transient sate of clathrate hydrates involved in the CH_4_-CO_2_ (pure/mixed) swapping process. For this, the following chemical reaction is considered to represent the transition between gas, water and hydrates as

1 $$g(G/A)+{n}_{H}w(A/I)\leftrightarrow h(H)$$

in which, *g* denotes the guest gas species, *n*_*H*_ the hydration number, *w* the water species and *h* the hydrate component. Four phases may coexist there, namely gas (*G*), aqueous (*A*), solid ice (*I*) and hydrate (*H*). With this, the following practical aspects are taken into account to characterize the hydrate phenomena:

- gas hydrates mostly occur in permeable marine sediments in presence of water (pure/with salt ions)^17^
- hydrates are likely to form in the interstitial pore space between porous particles^18,19^
- porous medium consists of irregular 3D particles with their uneven distribution
- pores of these particles are further irregular in size and shape
- these nanometer-sized pores also participate in hydrate formation and decay^1^. It is confirmed through the seismic survey studies conducted in the gas hydrate field of Alaska^20^, Blake Ridge^20,21^ and Mackenzie Delta^22^
- surface renewal is inevitable because of the barrier (i.e., solid hydrate film) grown during hydrate formation and decayed during dissociation at the interface^18^
- pure carbon dioxide and methane, and 3–20 mol% CO_2_ in air or with N_2_ gas form sI hydrate structure that consists of two small 5^12^ cages and six large 5^12^6^2^ cages per unit cell^23^, which is evident through PXRD pattern^5^
- pure and mixed CO_2_ (N_2_ major and CO_2_ minor) effectively act as the replacement agent. In fact, mixed CO_2_ has proven a better agent than pure CO_2_ ^4^ this is because CO_2_ gets preference to occupy large cages, and in small cages, CH_4_ and N_2_ compete to each other for better occupancy^24^. Thus, our formulation attempts to accommodate both options.
- along with sand, glass beads are used as the second porous medium that mimics the effect of sediments for sand, sandstone and kaoline clays^25^.

#### Activity

Activity of water (*a*_*w*_) has a great influence on hydrate dynamics. In the natural hydrate bearing atmosphere, it takes into account the collective effect of water in its pure form (subscript ‘*Pure*’), and in presence of salt ion (subscript ‘*SI*’) and porous medium (subscript ‘*PM*’) as

2 $${a}_{w}={a}_{w,Pure}+{a}_{w,SI}+{a}_{w,PM}$$

where, *a*_*w*, *Pure*_ = *γ*_*w*_*x*_*w*_, in which, *γ*_*w*_ represents the activity coefficient of water in water-gas mixture and x_w_ the concentration of that water. The estimation of *a*_*w*,*Pure*_ is briefly highlighted later. Further, the Pitzer model^26^ is employed to formulate the water activity in electrolytic solution as

3 $${a}_{w,SI}=\exp [-\,\frac{{\rm{MW}}}{1000}(\sum _{l}{m}_{l})\varphi ]$$

where, MW is the molecular weight of water and *m*_*l*_ the molality of solute species *l* (cation/anion/neutral). The osmotic coefficient, *ϕ* is estimated from^26^

4 $$\begin{array}{rcl}\varphi  & = & 1+\frac{2}{\sum _{l}{m}_{l}}[-\,\frac{{A}^{\varphi }{I}^{1.5}}{1+1.2{I}^{0.5}}+\sum _{c}\sum _{a}{m}_{c}{m}_{a}({B}_{ca}^{\varphi }+Z{C}_{ca})\\  &  & +\,\sum \sum _{c < c^{\prime} }{m}_{c}{m}_{c^{\prime} }({{\rm{\Phi }}}_{cc\text{'}}^{\varphi }+\sum _{a}{m}_{a}{\psi }_{cc^{\prime} a})\\  &  & +\,\sum \sum _{a < a^{\prime} }({{\rm{\Phi }}}_{aa^{\prime} }^{\varphi }+\sum _{c}{m}_{a}{\psi }_{aa^{\prime} c})+\sum _{n}\sum _{c}{m}_{n}{m}_{c}{\lambda }_{nc}]\\  &  & +\,\sum _{n}\sum _{a}{m}_{n}{m}_{a}{\lambda }_{na}+\sum _{n}\sum _{c}\sum _{a}{m}_{n}{m}_{c}{m}_{a}{\zeta }_{nca}\end{array}$$

in which, *I* is the ionic strength, *Z* is a function (see Methods section) and *A*^*ϕ*^ the one third of the Debye-Huckel limiting slope. Here, *B*^*ϕ*^, Φ^*ϕ*^, *λ* and *C*, *ψ*, *ζ* are the measurable combinations of the second and third virial coefficients, respectively. Note that the single summation indices, *c*, *a* and *n* denote the sum over all cations, anions and neutral ions in the system, respectively. The double summation indices, *c* < *c*′ and *a* < *a*′ refer to all the distinguishable pairs of dissimilar cations and anions, respectively.

The molality of salt ions is obtained from: *m*_*l*_ = *n*_*SI*_/*W*_*A*_, in which, *n*_*SI*_ denotes the moles of salt ions and *W*_*A*_ the amount of water remained in the aqueous phase, which is estimated from: *W*_*A*_ = *W*_*in*_(1 − *WC*). Here, *W*_*in*_ is the amount of water present prior to hydrate formation and *WC* the water conversion to hydrates during growth phase.

To formulate the activity of water in presence of porous medium, a couple of practical issues are to be addressed concerning the occurrence of gas hydrates in the irregular nanometer-sized pores with disordered capillaries of the distributed porous particles. Fractal theory^27^ is used to model the activity, *a*_*w*, *PM*_

5 $${a}_{w,PM}=\exp [-\,\frac{{V}_{w}}{RT}(\frac{2k}{{r}^{2-{D}_{f}}}\frac{{\sigma }^{\infty }}{(1+\frac{2k\delta }{{r}^{2-{D}_{f}}})})]$$

in which, *V*_*w*_ is the molar volume of water, *k* a constant, *D*_*f*_ the fractal dimension of the pore edge, *σ*^∞^ the interfacial energy between planar interfaces (0.0267 J.m^−2^)^28^, *δ* the Tolman length (0.4186 nm)^29^ and *R* the universal gas constant. This modeling equation is applicable to both the irregular capillaries and pore, and thus the radius, *r* corresponds to both the hydrate core and pore. Here, we propose *k* as a linear function of *r* as: *k* = *ar* + *b*; *a* and *b* are the coefficients, values of which can be determined from the experimental data^29^.

#### Driving Force

The driving force is proposed in terms of chemical potential (*μ*) that considers the combined effect of temperature, pressure and composition^19^. It is expressed as

6 $${\rm{\Delta }}\mu =\frac{{\mu }_{w}^{H}}{RT}-\frac{{\mu }_{w}^{L}}{RT}$$

where, ${\mu }_{w}^{H}$ and ${\mu }_{w}^{L}$ are the chemical potential of water in the filled hydrate and the liquid phase, respectively. Obviously, this Δ*μ* is positive for hydrate formation and negative for dissociation.

#### Interstitial Reaction Surface Area

As stated, clathrate hydrates mostly form in the interstitial pore space between porous materials when small guest molecules (<0.9 nm) contact water at high pressure and low temperature. To formulate the cage dynamics, one needs to first find the total surface area of the irregular 3D particles^30^ from: *A* = *eV*^2/3^, in which, *V* is the total volume of porous medium and *e* the scaling factor. For spherical shape, *e* is obviously 4.836 and for irregular solids, it is typically 6.2918. Now, the total volume of porous material is computed by subtracting the pore volume (*V*_*p*_) from bulk volume (*V*_*b*_) of the porous media with *V*_*b*_ = *m*/*ρ*_*b*_, where *m* and *ρ*_*b*_ denote the mass and bulk density of the porous material.

As forward reaction [in equation(1)] proceeds, the solid hydrate film starts increasing in size. Acting as a barrier at the interface, this film leads to decrease the contact area devoted for hydrate growth. Obviously, the reverse trend is true for backward reaction. Accordingly, the concept of effective surface area (*A*_*e*_) is introduced as: *A*_*e*_ = *βA*. As mentioned, the surface renewal is one of the important aspects of practical importance and it is proposed through updating the weighting factor as: *β*_0_ = *β*_0_ exp(*Ct*). Here, *β*_0_ denotes the surface area adjustment factor, and the constant, *C* is negative for hydrate formation and positive for dissociation.

#### Reaction Kinetics

Prior to formulating the transient swapping process, one should kinetically model the hydrate formation and dissociation, and then couple them with certain flexibility in their movement. For this, apart from the driving force, Δ*μ*, the hydrate kinetics is greatly influenced by the water consumption rate. With this, the reaction rate is formulated^12^ as

7 $$r=\frac{1}{{A}_{e}}\frac{d{n}_{g,H}}{dt}=({kn}_{{{\rm{H}}}_{{\rm{2}}}{\rm{O}},L})(\frac{{\mu }_{w}^{H}}{RT}-\frac{{\mu }_{w}^{L}}{RT})$$

In which,

8 $${n}_{{{\rm{H}}}_{{\rm{2}}}{\rm{O}},L}={n}_{{{\rm{H}}}_{{\rm{2}}}{\rm{O}},T}-{n}_{H}\sum _{i=1}^{{N}_{c}}{n}_{{g}_{i},H}={n}_{{{\rm{H}}}_{{\rm{2}}}{\rm{O}},T}-{n}_{H}{n}_{g,H}$$

and the rate constant^31^

9 $$k={k}_{0}\,\exp (\frac{-{\rm{\Delta }}E}{RT})$$

where, *n*_*g*,*H*_ denotes the moles of guest gas in hydrate phase, ${n}_{{{\rm{H}}}_{{\rm{2}}}{\rm{O}},L}$ the residual moles of water in liquid phase, ${n}_{{{\rm{H}}}_{{\rm{2}}}{\rm{O}},T}$ the total moles of water initially present, *k*_0_ the intrinsic rate constant, Δ*E* the activation energy, *N*_*c*_ the number of gas components and subscript *i* the component index.

#### Guest Gas Dynamics

Hydrate formation: For hydrate formation, the governing equation (7) yields

10 $$\frac{d{n}_{g,H}}{dt}={k}_{0}\,\exp (\frac{-{\rm{\Delta }}E}{RT})A{\beta }_{0}\,\exp \,(-\,Ct)({\rm{\Delta }}\mu )({n}_{{{\rm{H}}}_{{\rm{2}}}{\rm{O}},T}-{n}_{H}{n}_{g,H})$$

Integrating and then simplifying, one obtains

11 $${n}_{g,H}=\alpha \frac{{n}_{{{\rm{H}}}_{2}{\rm{O}},T}}{{n}_{H}}\{{\rm{1}}-\exp [-\frac{{n}_{H}{k}_{{\rm{0}}}{\beta }_{0}}{CRT}A\,\exp (\frac{-{\rm{\Delta }}E}{RT})({\mu }_{w}^{H}-{\mu }_{w}^{L})(1-\exp (-\,Ct))]\}$$

Here, *α* is a tuning parameter defined as the ratio of the highest amount of net guest gas consumed and the total amount of that gas ideally occupied in all cavities. This formulation will be used to represent the guest gas dynamics during hydrate formation and growth in salt water with porous media.

Hydrate dissociation: The dissociation kinetics is governed by

12 $$-\frac{d{n}_{g,H}}{dt}=k\,{A}_{e}\,{\rm{\Delta }}\mu \,{n}_{{{\rm{H}}}_{{\rm{2}}}{\rm{O}},H}$$

This is the modified form of equation (7), in which ${n}_{{{\rm{H}}}_{{\rm{2}}}{\rm{O}},H}$ denotes the moles of water in hydrate phase. Substituting all relevant terms and simplifying,

13 $${n}_{g,H}={n}^{0}\,\exp (\frac{{n}_{H}{k}_{{\rm{0}}}{\beta }_{0}}{\alpha RTC}A\,\exp \,(\frac{-{\rm{\Delta }}E}{RT})({\mu }_{{\rm{w}}}^{L}-{\mu }_{{\rm{w}}}^{H})\,(1-\exp (Ct)))$$

Here, *n*^0^ denotes the total moles of guest gas present in the hydrate phase. This is the final formulation for hydrate dissociation in porous media and saline environment.

Gas swapping: As stated, the swapping is a nondestructive process that proceeds with executing a dual mechanism of energy production and greenhouse gas sequestration. During this process, the pure/mixed CO_2_ is introduced in the existing CH_4_ hydrate bearing sediments at a reduced temperature and pressure to disturb the hydrate-gas equilibrium^32^. This leads to dissociating methane hydrates and forming mixed hydrates at the same time. At this point, it should be noted that methane and nitrogen gas compete to occupy mostly small cages, while carbon dioxide preferentially occupies large cages without any challenge from other guests^24^. The thermodynamic mechanism for guest molecule exchange is typically controlled by the prevailing temperature, pressure and component composition^33^, on which, the chemical potential truly depends. The replacement continues through hydrate formation along with dissociation until the transition of equation(1) reaches the equilibrium state (i.e., ${\mu }_{w}^{H}={\mu }_{w}^{L}$)^14^. This thermodynamic control mechanism can also be explained by estimating the changes in free energy of gas hydrates^33^.

With this complicacy, we formulate the swapping dynamics in terms of methane displacement rate as

14 $$\frac{d{n}_{{{\rm{CH}}}_{{\rm{4}}},H}}{dt}+{k}_{d}{A}_{e,d}{({\rm{\Delta }}\mu )}_{{{\rm{CH}}}_{{\rm{4}}}}{n}_{{{\rm{H}}}_{{\rm{2}}}{\rm{O}},H}=\xi {k}_{f}{A}_{e,f}{({\rm{\Delta }}\mu )}_{{\rm{RA}}}{n}_{{{\rm{H}}}_{{\rm{2}}}{\rm{O}},L}$$

The subscript *f* and *d* refer to the formation and dissociation of hydrate, respectively. Here, *ξ* is the ratio of fractional occupancy of CH_4_ to that of replacement agent (subscript ‘RA’) (i.e., pure/mixed CO_2_) in both small and large cages, which can be determined by the use of Langmuir type expression (equation (24)). Rewriting equation (14),

15 $$\begin{array}{rcl}\frac{d{n}_{{{\rm{CH}}}_{{\rm{4}}},H}}{dt} & = & -{k}_{d}{\beta }_{0}\exp (Ct)A{(\frac{{\mu }_{w}^{L}}{RT}-\frac{{\mu }_{w}^{H}}{RT})}_{{{\rm{CH}}}_{{\rm{4}}}}({n}_{H}{n}_{{{\rm{CH}}}_{{\rm{4}}},H})\\  &  & +\,\xi \,{k}_{f}{\beta }_{0}\exp \,(-\,Ct)\,A\,{(\frac{{\mu }_{w}^{H}}{RT}-\frac{{\mu }_{w}^{L}}{RT})}_{RA}({n}_{{{\rm{H}}}_{{\rm{2}}}{\rm{O}},T}-{n}_{H}{n}_{{{\rm{CH}}}_{{\rm{4}}},H})\end{array}$$

Using integrating factor method, one can get the following form

16 $$\begin{array}{rcl}{n}_{{{\rm{CH}}}_{{\rm{4}}},H} & = & \{\frac{\xi {k}_{f}{\beta }_{0}\,A{({\mu }_{w}^{H}-{\mu }_{w}^{L})}_{{\rm{RA}}}{n}_{{{\rm{H}}}_{{\rm{2}}}{\rm{O}},T}\,\exp (\frac{{k}_{d}{\beta }_{0}A{({\mu }_{w}^{L}-{\mu }_{w}^{H})}_{{{\rm{CH}}}_{{\rm{4}}}}{n}_{H}-\xi {k}_{f}{\beta }_{0}A{({\mu }_{w}^{H}-{\mu }_{w}^{L})}_{{\rm{RA}}}{n}_{H}}{RTC})}{RT\,\exp [(\frac{{k}_{d}{\beta }_{0}A}{RTC}{({\mu }_{w}^{L}-{\mu }_{w}^{H})}_{{{\rm{CH}}}_{{\rm{4}}}}{n}_{H}\,\exp \,(Ct))-(\frac{\xi {k}_{f}{\beta }_{0}A}{RTC}{({\mu }_{w}^{H}-{\mu }_{w}^{L})}_{{\rm{RA}}}{n}_{H}\,\exp \,(\,-Ct))]}\}\\  &  & \times \{\frac{\exp \{[(\frac{{k}_{d}{\beta }_{0}A}{RT}{({\mu }_{w}^{L}-{\mu }_{w}^{H})}_{{{\rm{CH}}}_{{\rm{4}}}}{n}_{H})+(\frac{\xi \,{k}_{f}{\beta }_{0}A}{RT}{({\mu }_{w}^{H}-{\mu }_{w}^{L})}_{{\rm{RA}}}{n}_{H})-C]\,t\}-1}{(\frac{{k}_{d}{\beta }_{0}A}{RT}{({\mu }_{w}^{L}-{\mu }_{w}^{H})}_{{{\rm{CH}}}_{{\rm{4}}}}{n}_{H})+(\frac{\xi \,{k}_{f}{\beta }_{0}A}{RT}{({\mu }_{w}^{H}-{\mu }_{w}^{L})}_{{\rm{RA}}}{n}_{H})-C}\}\\  &  & +\{\frac{{n}^{0}\,\exp (\frac{{k}_{d}{\beta }_{0}A\,{({\mu }_{w}^{L}-{\mu }_{w}^{H})}_{{{\rm{CH}}}_{{\rm{4}}}}{n}_{H}-\xi {k}_{f}{\beta }_{0}A\,{({\mu }_{w}^{H}-{\mu }_{w}^{L})}_{{\rm{RA}}}{n}_{H}}{RTC})}{\exp [(\frac{{k}_{d}{\beta }_{0}A}{RTC}{({\mu }_{w}^{L}-{\mu }_{w}^{H})}_{{{\rm{CH}}}_{{\rm{4}}}}{n}_{H}\,\exp \,(Ct))-(\frac{\xi \,{k}_{f}{\beta }_{0}A}{RTC}{({\mu }_{w}^{H}-{\mu }_{w}^{L})}_{{\rm{RA}}}{n}_{H}\,\exp \,(\,-Ct))]\,}\}\end{array}\,$$

This is the final theoretical representation of the proposed replacement kinetics that will be used in the sequel to analyze the transient swapping behavior of multicomponent gases with porous media and salt water.

### Comparison to experiments

The replacement process in hydrate-bearing sediments is really complex because of the formation of CH_4_-CO_2_ mixed hydrate and simultaneous dissociation of CH_4_ hydrate in a system consisting of four phases (i.e., porous medium, hydrate, water and natural gas). To describe the complex physicochemical phenomena, we would like to use the proposed theory through validating it by the real-time data for two different sets of experimental arrangements (see supplementary information). All model parameters are reported in Table S1. With this, to quantify the performance of the developed formulation, we have used the absolute average relative deviation (AARD).

#### CH_4_ – Pure CO_2_ Swapping with Aqueous Brine and Sand

First we study the CH_4_ replacement by pure CO_2_ (99.9 mol%) with the use of reported data^34^ for experimental setup 1 (see supplementary information). Clay layers are practically impermeable owing to compact packing between constituent particles^4^, whereas sand layers, which contain a significant amount of gas hydrates, are considered to be practically permeable. Therefore, we consider sandy-sediment to simulate a real NGH environment. For this, the aqueous brine used has a salinity of 3.35 wt% (Na_2_SO_4_) and the sediment is formed by 20–40 mesh quartz sands with a porosity of 38.7%.

**Figure 1:**
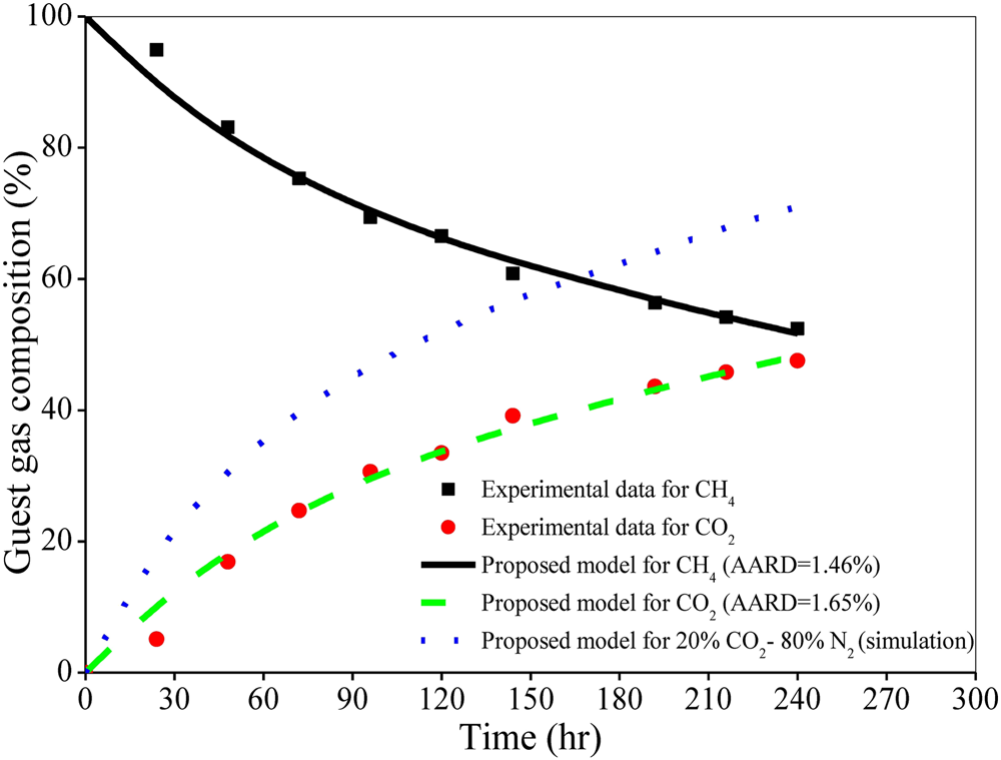
Performance of the proposed formulation with reference to experimental data^34^ for the compositional change of guest gases (CH_4_/CO_2_) in hydrate phase during replacement reaction. This reaction is performed at 3.18 MPa and 274.7 K by using pure CO_2_ gas in presence of aqueous brine and sand having particle size distribution of 420 to brine and sand having particle size distribution of 420 to 841 μm. The AARD (%) values used to quantify the model performance are provided in the figure.

The replacement reaction is performed in the reactor with 90.0 v% gas, 1.1 v% water and 8.9 v% hydrate saturation at stated operating pressure and temperature. During swapping, the natural gas gets replaced in hydrate cavities by the carbon dioxide gas. As a consequence, the CH_4_ concentration increases in the gas phase and decreases in the hydrate phase. Naturally, the concentration dynamics of CO_2_ is reverse in those two phases. This experiment leads to an about 45% of methane replacement by pure carbon dioxide. It is evident in Fig. 1 that the developed model shows a promising performance in predicting the swapping behavior between methane and carbon dioxide that is also confirmed through the AARD values.

Further, a direct comparison is made in Fig. 1 between CH_4_ swapping kinetics with CO_2_ (simulation and experiment) and CO_2_/N_2_ mixture (simulation only, since experimental data are not available). It is a fact that the density of injected CO_2_ is high, while its permeability is reasonably low. Adding N_2_ to CO_2_ leads to increase the gas permeability. Moreover, blocking of flow channels owing to the formation of new hydrate from injected CO_2_/N_2_ is less compared to injected pure CO_2_ ^35^. Mainly because of these reasons, the CO_2_ composition in hydrate phase is higher in case of CO_2_/N_2_ mixed guest gas compared to pure CO_2_, which is quite obvious in Fig. 1 with respect to time.

#### CH_4_ – Mixed CO_2_ Swapping with Glass Beads

In naturally occurring gas hydrate sites, injected carbon dioxide gas may transform to a liquid state at harsh conditions^5^. This leads to make injection and diffusion very unstable, yielding a low replacement rate and recovery. To overcome such weakness, the use of CO_2_/N_2_ gas mixture is proposed and successfully performed in a field production test on the Alaska North Slope in 2012^5^. This mixed gas improves the replacement efficiency compared to pure CO_2_ mainly because of replacing natural gas by CO_2_ in large cages and that by N_2_ in small cages^24^, along with the reasons mentioned in the last case study. Moreover, the formation condition of CO_2_ hydrate is known to be more stable than that of CH_4_ hydrate^31^, indicating the suitability of swapping process for long term CO_2_ storage^24^. Keeping these issues in mind, the proposed theory is tested here with the mixed CO_2_ gas as well in presence of glass beads that mimic the effect of sediments for sand, sandstone and kaoline clays, which have little to no swelling in contact with water^25^.

**Figure 2:**
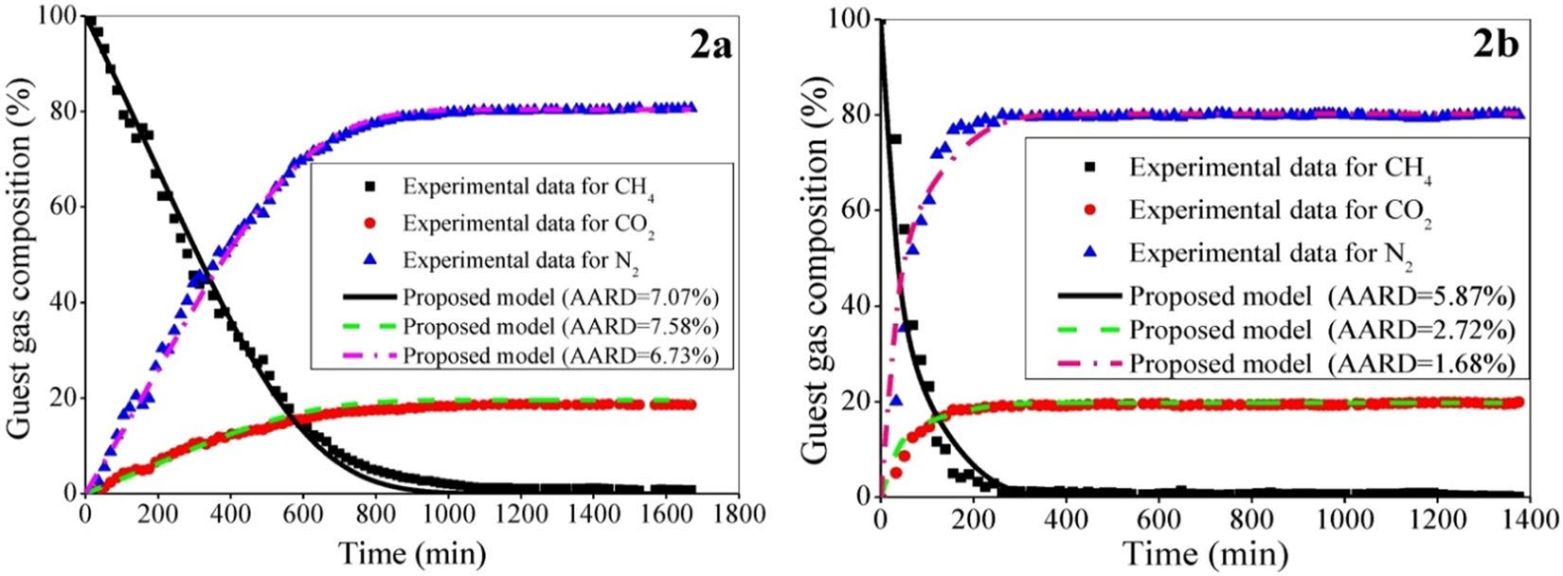
Performance of the proposed formulation with reference to reported data^4^ for experimental setup 2 for the compositional change of multicomponent guest gas (CH_4_/CO_2_/N_2_) in hydrate phase during replacement reaction. It is performed at 9.8 MPa and 275.15 K in the reactor packed with glass beads of 100 μm average size with a porosity of 32.1%. The gas mixture of CO_2_-N_2_ is injected at a rate of (**a**) 100 sccm and (**b**) 200 sccm. The experimental data is collected from the first sampling port situated at 0.7 m from the inlet of the reactor.

With this, the performance of the developed formulation is evaluated with simulating the compositional dynamics at a distance of 0.7 m from the inlet of the 8 m long reactor. Here, the CH_4_ present in hydrate cavities is attempted to swap by injecting the replacement gas mixture (i.e., 20 mol% CO_2_ and 80 mol% N_2_) into the reactor at a rate of 100 sccm (Fig. 2a) and 200 sccm (Fig. 2b). The replacement reaction starts in the reactor with 73.67 v% gas, 14.6 v% water and 11.73 v% hydrate saturation for 100 sccm case, and 73.64 v% gas, 14.6 v% water and 11.76 v% hydrate saturation for 200 sccm case. The reactor contains a porous bed formed with glass beads.

The guest gas (i.e., CO_2_/N_2_ mixture) injection rate truly affects the replacement kinetics and total run time^4^. For example, increasing gas injection rate leads to shallow penetration into the gas hydrates layers. It is because of short residence time with high flux, and thereby low replacement efficiency. On the other hand, the low flux of the guest gas leads to deep penetration and more extended stay, which results in improved replacement efficiency. Here, we have demonstrated the model prediction for two different gas mixture injection rates (i.e., 100 and 200 sccm). Due to the stated reason and as experimentally observed^4^, the 200 sccm case (Fig. 2b) leads to decrease the replacement efficiency compared to the case with 100 sccm injection rate (Fig. 2a). Further, the run time decreases with increasing injection rate as the methane gets depleted rapidly in the reactor^4^. It is obvious that the proposed model provides a reasonably good prediction of swapping data.

Injecting guest gas (e.g., CO_2_) into *in-situ* natural gas hydrates in sediments leads to conversion over to CO_2_ and mixed CO_2_/CH_4_ hydrates. Now, this conversion is governed by the two primary mechanisms^35^. They are based on: (i) direct solid state conversion, and (ii) formation of new hydrates from injected guest gas and free water. In the first mechanism, the CH_4_ hydrate directly converts into pure CO_2_ and mixed CO_2_/CH_4_ hydrate. This is a very slow process because of slow mass transport through hydrate and it dominates only when there is no sufficient free water available. The previous case study (Fig. 1) deals with only 1.1 v% water saturation, and thus the first mechanism dominates there, which leads to reach about 20% CO_2_ composition in hydrate phase taking a reasonably long time (around 60 hr).

As far as the second mechanism is concerned, the injected CO_2_ gas reacts with free water in the porous media and forms new CO_2_ hydrate. This reaction is exothermic in nature and thus, it releases heat, which in turn further dissociates the surrounding CH_4_ hydrate. Then, with the generated water, the CO_2_ forms more hydrates. Note that this mechanism dominates if sufficient amount of free water is available there and it is faster than the direct solid state exchange mechanism. The CO_2_ gas consumption from the mixed CO_2_/N_2_ guest gas in Fig. 2 follows this mechanism (14.6 v% water saturation) and thus, it reaches about 20% CO_2_ composition in around 10 hr, which is about 60 hr in the previous case (1.1 v% water saturation) (Fig. 1).

**Figure 3:**
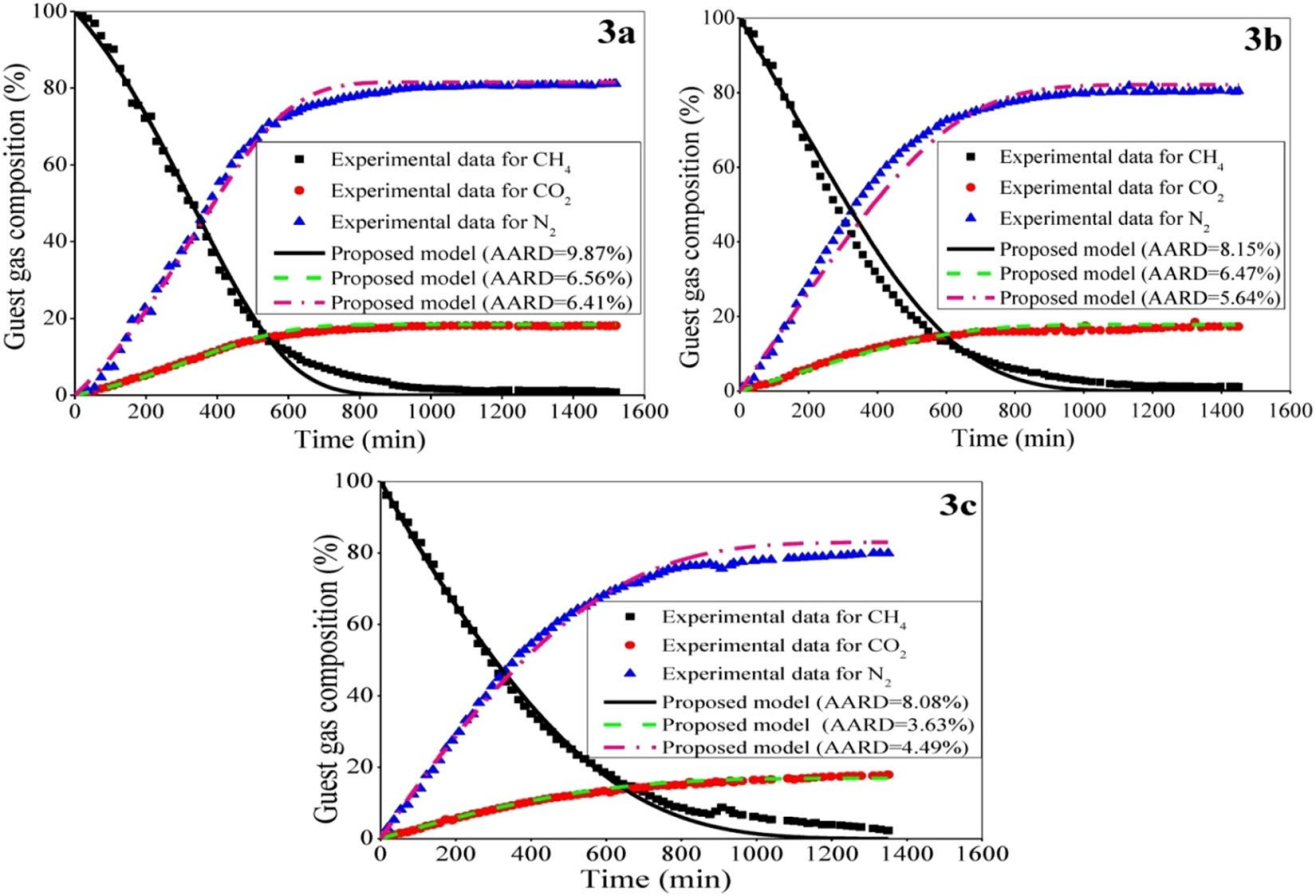
Performance of the proposed formulation with reference to experimental data^4^ for the compositional change of multicomponent guest gas (CH_4_/CO_2_/N_2_) in hydrate phase at a distance of (**a**) 2.4 m, (**b**) 4 m and (**c**) 5.6 m from the inlet of the reactor during replacement reaction. The reactor packed with glass beads is performed at 9.8 MPa and 275.15 K with a 100 sccm injection rate of the gas mixture of CO_2_-N_2_.

In the subsequent study, the guest exchange ability of CO_2_/N_2_ mixture is shown at the prevailing condition by attacking and replacing the encaged CH_4_ in hydrate bearing sediments. The comparative gas composition profiles are produced as a function of length for an injection rate of 100 sccm, keeping all other conditions same with the last test (i.e., pressure = 9.8 MPa, temperature = 275.15 K, replacement agent having 20 mol% CO_2_ and 80 mol% N_2_, 100 μm average size glass beads, and 14.6 v% water saturation). With this, Fig. 3 compares the proposed model predictions with reference to the experimental data^4^ for three different sampling ports situated at (a) 2.4 m, (b) 4 m and (c) 5.6 m in the 8 m long one-dimensional tubular reactor. Expectedly, the CH_4_ composition decreases with time, and for CO_2_ and N_2_, the trend is just reverse. As the guest gas is injected, the CH_4_ is displaced, triggering spontaneous swapping reaction in the hydrate phase.

This test further proves that the proposed formulation is capable of predicting the replacement phenomena with reasonable accuracy that is indicated through the AARD values. It should be noted that for establishing the flow-front of gases in the sandy gas hydrate layer and a reliable estimation of natural gas recovery, this integrated dynamic compositional information along the length is inevitable^4^.

## Discussion

Gas swapping inside the hydrate lattice is proposed as a nondestructive process that can provide the next generation energy for a couple of centuries and reduce the emission of greenhouse gas arising from anthropogenic activities. Addressing several fundamental features of gas hydrates and their occurrence in permafrost and under deep seabed at diverse geological conditions in theory, attempt is made to make the model realistic and generalized enough. Two porous media composed of unevenly distributed sand particles and glass beads are separately used to investigate the model performance in presence of pure water and aqueous brine. There is a key finding reported through the experimental investigation that CH_4_-CO_2_/N_2_ provides a higher replacement efficiency than CH_4_-CO_2_ ^4^. Therefore, the proposed formulation is tested with binary and multicomponent guest gases to show its versatility. With this, the developed formulation describes the swapping phenomena precisely and provides a promising performance with a close agreement with the experimental data.

This formulation can further be used

- to understand the hydrate characteristics involved in natural gas storage and transportation, desalination, gas separation, and CO_2_ capture and sequestration, among others
- as a scale-up model for all the above-mentioned hydrate specific processes

Providing clearer insight into how hydrates are generated, deposited and decayed, this model can play a crucial role in the assessment of total gas reserves in hydrate deposits that is still a highly volatile issue, involving a wide range of uncertainty.

## Methods

### Activity

#### Finding a_w,Pure_

First of all, we need to determine *x*_*w*_ from: *x*_*w*_ = 1−*x*_*g*_, when *γ*_*w*_ for pure water is unity. Then one can obtain the composition of guest gas (*x*_*g*_) by knowing the molality of that gas (*m*_*g*_) from

17 $${m}_{g}={y}_{g}{P}_{T}\,\exp (\frac{{\mu }_{g}^{V(0)}-{\mu }_{g}^{L(0)}}{RT}+\,\mathrm{ln}\,{\varphi }_{g}-\,\mathrm{ln}\,{\gamma }_{g})$$

For the guest gas, *μ*^*L*(0)^ and *μ*^*V*(0)^ are the standard chemical potential in the liquid and vapor phase, respectively, and *ϕ* the fugacity coefficient that is obtained with the use of Soave-Redlich-Kwong (SRK) equation of state^36^. Here, *μ*^*V*(0)^ is adopted as zero^37^, and *μ*^*L*(0)^ is considered as a function of operating temperature and pressure^37^.

#### Finding a_w,SI_

The parameters, namely *A*^*ϕ*^, *C*^*ϕ*^ and *ψ*, are function of temperature (*T*) as

18 $$f(T)={c}_{1}+{c}_{2}T+\frac{{c}_{3}}{T}+{c}_{4}\,\mathrm{ln}\,T+{c}_{5}{T}^{2}+{c}_{6}{T}^{3}$$

whereas, *λ* and *ζ* are dependent on temperature (*T*) and pressure (*P*) as

19 $$f(T,P)={C}_{1}+{C}_{2}T+\frac{{C}_{3}}{T}+{C}_{4}\frac{P}{T}+{C}_{5}\frac{{P}^{2}}{T}+{C}_{6}\,T\,\mathrm{ln}\,P$$

Values of constants, *c*_1_ to *c*_6_ and *C*_1_ to *C*_6_, for all these five parameters are available in literature^26^. Further, the ionic strength (*I*) is defined as: $I=\frac{1}{2}\sum _{l}{m}_{l}{z}_{l}^{2}$, where *z*_*l*_ is the charge of ion *l*. Moreover, equation (4) includes *Z* that is expressed as: $Z=\sum _{l}{m}_{l}|{z}_{l}|$.

Second virial coefficient, ${B}_{ca}^{\varphi }$ has the following representation

20 $${B}_{ca}^{\varphi }={\beta }_{ca}^{(0)}+{\beta }_{ca}^{(1)}{e}^{-{\alpha }_{ca}\sqrt{I}}+{\beta }_{ca}^{(2)}{e}^{-12\sqrt{I}}$$

where, *β*^(0)^, *β*^(1)^and *β*^(2)^ are the temperature dependent parameters and equation (18) is used to calculate them. Note that *α*_*ca*_ is equal to 2.0 for univalent and 1.4 for higher valence pairs^26^. Besides, an another second virial coefficient, Φ that accounts for the interaction between the ions of equal sign (*l*−*m*), is given as

21 $${{\rm{\Phi }}}_{lm}^{\varphi }={{\rm{\Theta }}}_{lm}+{}^{E}{\rm{\Theta }}_{lm}(I)+{I}^{E}{{\rm{\Theta }}^{\prime} }_{lm}(I)$$

Here, Θ is a single parameter for each pair of anions or cations, and the functions, ^*E*^Θ_*lm*_(*I*) and ^*E*^Θ′_*lm*_(*I*), take into account the electrostatic unsymmetrical mixing effect and depend on ionic strength and the type of electrolyte pair. The single electrolyte third virial coefficient, *C*_*ca*_ is estimated as: ${C}_{ca}={C}_{ca}^{\varphi }/2\sqrt{|{z}_{c}{z}_{a}|}$.

#### Finding a_w,PM_

It is governed by the following equation

22 $${a}_{w,PM}=\exp (\frac{{V}_{w}(-\,{\rm{\Delta }}P)}{RT})$$

where, Δ*P* is the difference in pressure between the liquid and hydrate phase. The surface effects of the pore edge of irregular capillaries and irregular pores on Δ*P* are taken care of through: ${\rm{\Delta }}P=\frac{p}{s}{\sigma }_{H-W}\,\cos \,\theta$, in which, *p* is the perimeter (=2*πkr*^*Df*^), *s* the area (=*πr*^2^) and *θ* the wetting angle between hydrate and pore wall, which is zero^29^. Further, the surface tension between liquid water and hydrate is expressed as: *σ*_*H*−*W*_ = *σ*^∞^/1 + *κδ*, in which, the solid-liquid interface curvature, $\kappa =2k/{r}^{2-{D}_{f}}$.

### Driving force

The chemical potential of water in filled hydrate cages has the following form

23 $${\mu }_{w}^{H}={\mu }_{w}^{0}-{\rm{\Delta }}\,{\mu }_{w}^{H}$$

where, ${\mu }_{w}^{0}$ is the chemical potential of water in empty hydrate cages, ${\rm{\Delta }}\,{\mu }_{w}^{H}$ the difference in chemical potential of water between empty and filled hydrate cages in hydrate phase, which is calculated from^31,38^: ${\rm{\Delta }}{\mu }_{w}^{H}=-\,RT\,[\sum _{n=1}^{2}{v}_{n}\,\mathrm{ln}\,(1-\sum _{i=1}^{{N}_{{\rm{c}}}}{\theta }_{ni})]$. Here, *ν*_*n*_ is the number of cavities or cages of type *n* per water molecule in the crystal lattice. Further, the fraction of *n*th cavity occupied by *i*th guest molecule, *θ*_*ni*_ is

24 $${\theta }_{ni}=(\frac{{C}_{ni}\,{f}_{i}}{1+\sum _{i=1}^{{N}_{c}}{C}_{ni}\,{f}_{i}})$$

The fugacity of guest *i* in the hydrate phase (*f*_*i*_) is estimated from the SRK equation of state and it is assumed same with that of the gas phase^39^. The Langmuir constant is computed from^38^: ${C}_{ni}=\frac{4{\rm{\pi }}}{KT}\,{\int }_{0}^{R}\exp (\frac{-\omega (r)}{KT}){r}^{2}{\rm{d}}r$. Here, *K* denotes the Boltzmann’s constant, *R* the cell radius of hydrate and *ω*(*r*) the spherically symmetric cell potential. With this, the fractional occupancy of CH_4_ to replacement agent (subscript ‘RA’) in both small (subscript ‘S’) and large (subscript ‘L’) cages during replacement is represented for equation (14) as: $\xi =({\theta }_{{\rm{S}},{{\rm{CH}}}_{{\rm{4}}}}+{\theta }_{{\rm{S}},{{\rm{CH}}}_{{\rm{4}}}})/({\theta }_{{\rm{S}},{\rm{RA}}}+{\theta }_{{\rm{S}},{\rm{RA}}})$.

Like ${\mu }_{w}^{H}$, ${\mu }_{w}^{L}$ includes ${\mu }_{w}^{0}$ and ${\rm{\Delta }}\,{\mu }_{{\rm{w}}}^{{\rm{L}}}$ according to equation (23). This ${\rm{\Delta }}\,{\mu }_{{\rm{w}}}^{{\rm{L}}}$, which represents the difference in chemical potential of water between the empty hydrate cages in hydrate phase and the liquid phase, is estimated from

25 $$\frac{{\rm{\Delta }}{\mu }_{w}^{L}(T,P)}{RT}=\frac{{\rm{\Delta }}{\mu }_{w}^{0}(T,0)}{R{T}_{0}}-{\int }_{{T}_{0}}^{T}\frac{{\rm{\Delta }}{h}_{w}^{L}(T)}{R{T}^{2}}\,dT+{\int }_{0}^{P}\frac{{\rm{\Delta }}{V}_{w}^{L}}{R{T}^{2}}dP-\,\mathrm{ln}({a}_{w})$$

in which, *T*_0_ is the reference temperature, ${\rm{\Delta }}\,{\mu }_{{\rm{w}}}^{{\rm{0}}}(T,\,0)$ the standard chemical potential difference of water for gas hydrate at reference temperature and absolute zero pressure, ${\rm{\Delta }}\,{V}_{w}^{{\rm{L}}}$ the difference between molar volume of water in hydrate and liquid phase, and ${\rm{\Delta }}\,{h}_{w}^{{\rm{L}}}$ and ${\rm{\Delta }}\,{C}_{p}^{{\rm{L}}}$ the enthalpy and heat capacity difference between empty hydrate cages and liquid water, respectively.

### Absolute average relative deviation (AARD)

It is defined as: %${\rm{AARD}}=\frac{{\rm{100}}}{{n}_{dp}}\sum _{j=1}^{n}|\frac{{x}_{g,e}-{x}_{g,p}}{{x}_{g,e}}|$, in which, *n*_*dp*_ is the number of data points, and *x*_*g*,*e*_ and *x*_*g*,*p*_ the experimental and model predicted guest gas composition, respectively.

## Electronic supplementary material

Supplementary Information
