# Supplementary material for: Fundamental of swapping phenomena in naturally occurring gas hydrates
Source: Sci Rep. 2018 Nov 8;8:16563. doi: 10.1038/s41598-018-34926-2 (PMC6224528; doi:10.1038/s41598-018-34926-2)
Supplement: Supplementary file 1 — Supplementary Information [file 41598_2018_34926_MOESM1_ESM.pdf]

Fundamental of swapping phenomena in naturally occurring gas hydrates

Avinash V. Palodkar and Amiya K. Jana

Energy and Process Engineering Laboratory, Department of Chemical Engineering, Indian Institute of Technology, Kharagpur, 721302, India. Correspondence and requests for materials should be addressed to A.K.J. (email: akjana@che.iitkgp.ac.in)

Supplementary Information

SUPPLEMENTARY TEXT

Experimental arrangement: setup 1 (*34*)

As shown in Fig. S1, the reactor has an inner diameter of 300 mm with an effective height of 100 mm. There is a porous stainless steel sheet used to separate the upper sediments from the underlying free gas room in the reactor. Note that the mixture of sand and brine is only charged into the upper space at the beginning. Pressure transducers are used to monitor the pressure in both sides. Total sixteen thermocouples are employed and for temperature control, the reactor is immersed in a water bath system that contains ethylene glycol. A gas chromatogram (HP6890) is installed to measure the composition.

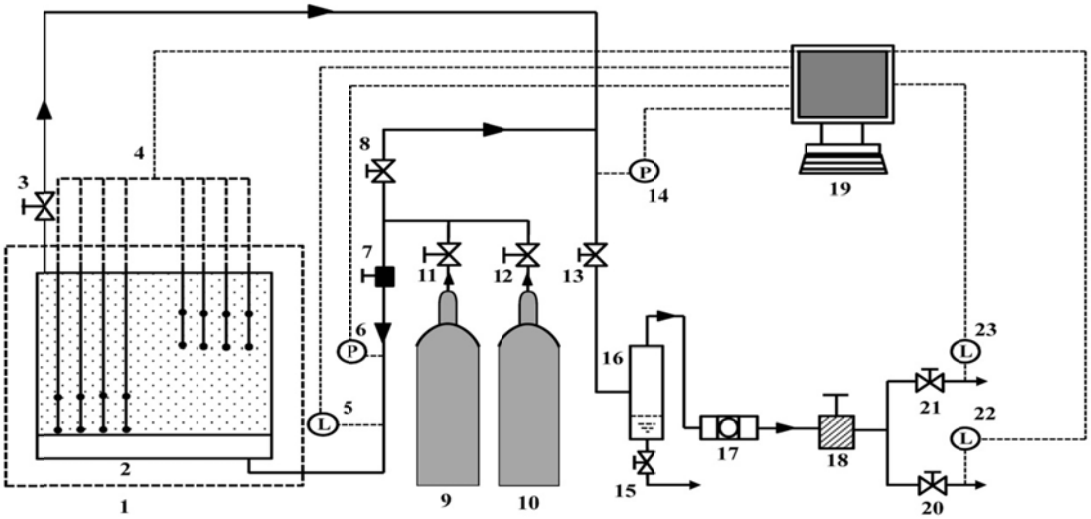

Fig. S1. Schematic of experimental arrangement (setup 1) for CH_4-CO_2 swapping (*34*). 1, water bath; 2, reactor; 3,7,8,11,12,13,15,20,21, valve; 4, thermocouple temperature transducer; 5,22,23, gas mass flow transducer; 6,14, pressure transducer; 9, methane cylinder; 10, carbon dioxide cylinder; 16, gas-water separator; 17, filter; 18, back-pressure regulator; 19, computer.

Experimental arrangement: setup 2 (*4*)

As shown the experimental setup in Fig. S2, the high pressure vessel is considered reasonably large in size (ID = 1.65 cm, OD = 1.905 cm, total length = 8 m). It is made large mainly to evaluate the actual aspects of replacement reaction taking place in NGH reservoir conditions and shown through experiments that a reasonable length (2.4 – 5.6 m) is truly needed to achieve a noticeable recovery rate. There are total 5 ports installed to collect the guest gas samples at 0.7, 2.4, 4.0, 5.6 and 7.3 m from the inlet of the reactor. An online gas chromatography is used for composition measurement. The 4-wire PT-100 thermocouple probe and pressure transducer are there at the sampling ports. Desired temperature is maintained by an external chiller through glycol bath.

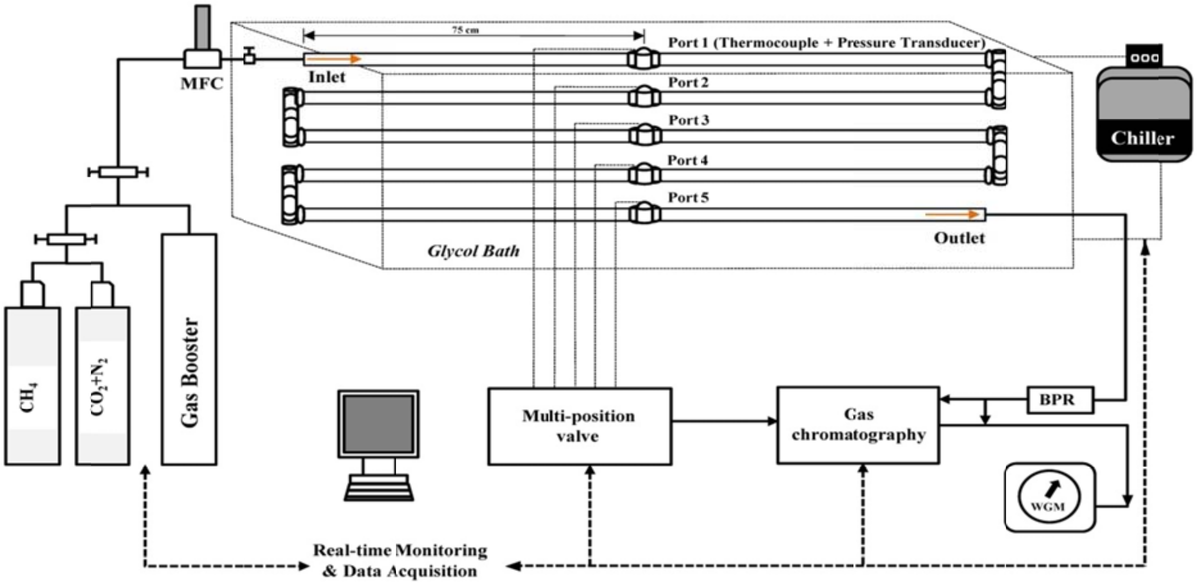

Fig. S2. Schematic of experimental arrangement (setup 2) for CH_4-CO_2/N_2 swapping (*4*). BPR – back pressure regulator, MFC – mass flow controller.

SUPPLEMENTARY TABLE

Table S1. Model parameters. The swapping formulation involves total three adjustable parameters, namely k_0, β_0 and C, and they all are tuned by the generalized reduced gradient (GRG) nonlinear optimization technique.

Fig.	Replacement agent	Parameter		
		$k_0 \times 10^{-13}$ (mol guest gas. mol H_2O^{-1}.m^{-2}.min^{-1})	β_0	$C \times 10^3$
1	CO_2 (99.9 mol%)	0.2982	0.7980	0.0223
2 (a)	CO_2-N_2 (20-80 mol%)	54.9604	0.2839	2.3382
2 (b)	CO_2-N_2 (20-80 mol%)	26.8440	0.9845	3.5257
3 (a)	CO_2-N_2 (20-80 mol%)	9.5509	0.4678	3.6946
3 (b)	CO_2-N_2 (20-80 mol%)	9.3025	0.5272	2.9014
3 (c)	CO_2-N_2 (20-80 mol%)	7.1017	0.6186	1.5489

.....
